# Detection and functional assessment of regulatory T cells in clinical samples

**DOI:** 10.1186/2051-1426-2-S3-P154

**Published:** 2014-11-06

**Authors:** Saskia Santegoets, Marij JP Welters, Sjoerd H van der Burg

**Affiliations:** 1 Department of Clinical Oncology, Leiden University Medical Center, Netherlands; 2 Leiden University Medical Center, Leiden, Netherlands

## 

Regulatory T cell (Treg)-mediated immunosuppression is considered a major obstacle for successful cancer immunotherapy. Given their profound effect on the outcome of immunotherapy trials, Treg frequency and function are being studied extensively. Unfortunately, no consensus has been reached about a) the (minimal number of) markers required to define human Tregs and b) which assay is the best to measure Treg suppressor function.

We had organized a workshop on the detection and functional testing of (antigen-specific) Tregs. Based on the outcome of this workshop and subsequent discussions, we proposed the following essential marker set for flow cytometric Treg analysis: CD3, CD4, CD25, CD127, FoxP3, Ki67 and CD45RA. We have validated the use of this essential marker set in a series of PBMC from healthy donors and cancer patients, as well as in tumor draining lymph nodes and freshly isolated tumors. We here show that the CD3, CD4, CD25, CD127 and FoxP3 markers are the minimally required markers to define human Treg cells in peripheral blood, tumors and lymph nodes. Importantly, as Ki67 and CD45RA provide additional information on their activation status, we recommend to also include these markers in the essential Treg marker set.

Similarly, the discussion on functional assays revealed that classical suppressor cell assays measuring the inhibition of responder cell proliferation or cytokine production in 3-7 day long assays required large numbers of Tregs and were technically challenging. Other assays, in particular an assay measuring Treg-mediated prevention of CD69 and CD154 up-regulation on CD4+ T cells was used as alternative and short (6-20h) assay for assessing human Treg suppressor function. We studied the ability of Tregs to inhibit CD25, CD69, CD154 and CD137 up-regulation on CD4+ and CD8+ T cells and compared this to the classical assay. Indeed Tregs were able to inhibit aCD3/CD28-bead-induced up-regulation of CD25, CD69 (on CD4 and CD8) and CD154 (on CD4), but not CD137 (on CD8) on responder T cells. Moreover, we showed that this excellently correlated with suppression of T cell proliferation.

In conclusion, we have identified CD3, CD4, CD25, CD127, FoxP3, Ki67 and CD45RA as the minimally required marker to define Tregs and showed that the T cell activation marker assay is a simple, fast and robust assay to measure Treg suppressive activity. The proposed essential marker set will be used to launch proficiency panels and harmonize the phenotypic analysis of Tregs within the CIMT Immunoguiding Program (CIP) participating laboratories.

